# Rates of Latent Tuberculosis in Health Care Staff in Russia

**DOI:** 10.1371/journal.pmed.0040055

**Published:** 2007-02-13

**Authors:** Francis Drobniewski, Yanina Balabanova, Elena Zakamova, Vladyslav Nikolayevskyy, Ivan Fedorin

**Affiliations:** 1 Health Protection Agency Mycobacterium Reference Unit and Clinical TB and HIV Group, Institute of Cell and Molecular Sciences, Barts and The London School of Medicine and Dentistry, Queen Mary College, University of London, London, United Kingdom; 2 Samara Oblast Tuberculosis Service, Samara, Russian Federation; Médecins Sans Frontières, The Netherlands

## Abstract

**Background:**

Russia is one of 22 high burden tuberculosis (TB) countries. Identifying individuals, particularly health care workers (HCWs) with latent tuberculosis infection (LTBI), and determining the rate of infection, can assist TB control through chemoprophylaxis and improving institutional cross-infection strategies. The objective of the study was to estimate the prevalence and determine the relative risks and risk factors for infection, within a vertically organised TB service in a country with universal bacille Calmette-Guérin (BCG) vaccination.

**Methods and Findings:**

We conducted a cross-sectional study to assess the prevalence of and risk factors for LTBI among unexposed students, minimally exposed medical students, primary care health providers, and TB hospital health providers in Samara, Russian Federation. We used a novel in vitro assay (for gamma-interferon [IFN-γ]) release to establish LTBI and a questionnaire to address risk factors. LTBI was seen in 40.8% (107/262) of staff and was significantly higher in doctors and nurses (39.1% [90/230]) than in students (8.7% [32/368]) (relative risk [RR] 4.5; 95% confidence interval [CI] 3.1–6.5) and in TB service versus primary health doctors and nurses: respectively 46.9% (45/96) versus 29.3% (34/116) (RR 1.6; 95% CI 1.1–2.3). There was a gradient of LTBI, proportional to exposure, in medical students, primary health care providers, and TB doctors: respectively, 10.1% (24/238), 25.5% (14/55), and 55% (22/40). LTBI was also high in TB laboratory workers: 11/18 (61.1%).

**Conclusions:**

IFN-γ assays have a useful role in screening HCWs with a high risk of LTBI and who are BCG vaccinated. TB HCWs were at significantly higher risk of having LTBI. Larger cohort studies are needed to evaluate the individual risks of active TB development in positive individuals and the effectiveness of preventive therapy based on IFN-γ test results.

## Abstract 2

Gamma-interferon assays were used in a cross-sectional study of Russian health care workers and found high rates of latent tuberculosis infection.

## Editors' Summary

**Background.:**

Tuberculosis (TB) is a very common and life-threatening infection caused by a bacterium, *Mycobacterium tuberculosis,* which is carried by about a third of the world's population. Many people who are infected do not develop the symptoms of disease; this is called “latent infection.” However, it is important to detect latent infection among people in high-risk communities, in order to prevent infected people from developing active disease, and therefore also reduce the spread of TB within the community. 22 countries account for 80% of the world's active TB, and Russia is one of these. Health care workers are particularly at risk for developing active TB disease in Russia, but the extent of latent infection is not known. In order to design appropriate measures for controlling TB in Russia, it is important to know how common latent infection is among health care workers, as well as other members of the community.

**Why Was This Study Done?:**

The researchers here had been studying the spread of tuberculosis in Samara City in southeastern Russia, where the rate of TB disease among health care workers was very high; in 2004 the number of TB cases among health care workers on TB wards was over ten times that in the general population. There was also no information available on the rates of latent TB infection among health care workers in Samara City. The researchers therefore wanted to work out what proportion of health care workers in Samara City had latent TB infection, and particularly to compare groups whom they thought would be at different levels of risk (students, clinicians outside of TB wards, clinicians on TB wards, etc.). Finally, the researchers also wanted to use a new test for detecting latent TB infection. The traditional test for detecting TB infection (tuberculin skin test) is not very reliable among people who have received the Bacillus Calmette-Guérin (BCG) vaccination against TB earlier in life, as is the case in Russia. In this study a new test was therefore used, based on measuring the immune response to two proteins produced by M. tuberculosis, which are not present in the BCG vaccine strain.

**What Did the Researchers Do and Find?:**

In this study the researchers tested health care workers from all the TB clinics in Samara City, as well as other clinical staff and students, for latent tuberculosis. In total, 630 people had blood samples taken for testing. A questionnaire was also used to collect information on possible risk factors for TB. As expected, the rate of latent TB infection was highest among clinical staff working in the TB clinics, 47% of whom were infected with M. tuberculosis. This compared to a 10% infection rate among medical students and 29% infection rate among primary care health workers. The differences in infection rate between medical students, primary care health workers, and TB clinic staff were statistically significant and reflected progressively increasing exposure to TB. Among primary care health workers, past exposure to TB was a risk factor for having latent TB infection.

**What Do These Findings Mean?:**

This study showed that there was a high rate of latent TB infection among health care workers in Samara City and that infection is increasingly likely among people with either past or present exposure to TB. The results suggest that further research should be carried out to test whether mass screening for latent infection, followed by treatment, will reduce the rate of active TB disease among health care workers and also prevent further spread of TB. There are concerns that widespread treatment of latent infection may not be completely effective due to the relatively high prevalence of drug-resistant TB strains and any new initiatives would therefore need to be carefully evaluated.

**Additional Information.:**

Please access these Web sites via the online version of this summary at http://dx.doi.org/10.1371/journal.pmed.0040055.

- The Stop TB Partnership has been set up to eliminate TB as a public health problem; its site provides data and resources about TB in each of the 22 most-affected countries, including Russia
- Tuberculosis minisite from the World Health Organization, providing data on tuberculosis worldwide, details of the Stop TB strategy, as well as fact sheets and current guidelines
- The US Centers for Disease Control has a tuberculosis minisite, including a fact sheet on latent TB
- Information from the US Centers for Disease Control about the QuantiFERON-TB Gold test, used to test for latent TB infection in this study

## Introduction

Identifying individuals with latent tuberculosis infection (LTBI) (considered to be persistence and multiplication of viable Mycobacterium tuberculosis bacilli within macrophages but without clinical manifestation of disease [1]) and their subsequent chemoprophylaxis is an important measure in preventing the development of active tuberculosis (TB), which can help to reduce the overall incidence and spread of TB.

Russia is one of the 22 World Health Organization-defined high-incidence TB countries; high rates of drug resistance, including multi-drug resistance have been reported from several regions [2–4].

Bacille Calmette-Guérin (BCG) vaccination is national policy in Russia and is administered to newborn babies and then repeated at 7 and 14–15 years of age [5]. The repetitive administration of BCG (and possibly also hypersensitisation from multiple annual tuberculin [PPD] screens) makes diagnosis of LTBI using traditional tuberculin skin tests (TSTs) in Russia challenging.

Similarly, health care workers (HCWs), particularly those working with patients with TB, are at a high risk of developing TB as a consequence of the vertical TB management programme (the TB service is distinct from the pulmonology and infectious disease services), prolonged hospitalisation of TB patients, the lack of isolation facilities, and weak infection control measures.

The general TB incidence rate in Samara Oblast in 2004, a region in Russia with a population of 3.3 million (Samara City, the capital has a population of 1.3 million), was 69.3/100,000, but TB incidence among TB staff was 741.6 per 100,000 [6]. No information is available for rates of LTBI in this group, largely due to the methodological problems associated with interpreting TST in a population that is universally immunised for BCG (commonly twice) and will have had multiple annual PPD examinations throughout childhood. High rates of LTBI would support the need for the review and reinforcement of institutional cross-infection measures.

Novel tests for latent infection have been developed based on the measurement of gamma-interferon (IFN-γ) production from peripheral blood mononuclear cells in response to two M. tuberculosis secreted proteins, ESAT-6 and CFP-10, absent in the vaccine strain BCG [7,8]. Two commercial systems (QuantiFERON–TB Gold, Cellestis, http://www.cellestis.com and T-Spot; Oxford Immunotec, http://www.oxfordimmunotec.com) have been used and reported to provide more sensitive and specific detection of latent and subclinical forms of TB than do traditional TSTs [7,9]. However, these systems have not been extensively tested for detection of LTBI in HCWs in a high TB incidence region against a background of universal BCG vaccination.

The aim of this study was to estimate the prevalence of latently infected clinical staff within the TB service (high-exposure group), in comparison to students prior to their employment (low-exposure group), and other clinical staff not primarily working with TB patients (medium exposure), and to test our hypothesis that infected cases increase in proportion to increasing occupational exposure to TB. Furthermore, we aimed to determine the relative risks and risk factors for infection.

## Methods

We conducted a comprehensive cross-sectional study of LTBI amongst clinical staff and students from October 2004 until October 2005 in Samara City. The prevalence of LTBI was established in three exposure groups: low (medical students from Samara Medical University [*n* = 238] and nonmedical students [*n* = 130] from Samara State University), intermediate (primary care physicians and nurses), and high (TB service doctors and nurses). Each of the participants gave written informed consent. The study was approved by the ethics committee of Samara State Medical University. Each participant completed a structured questionnaire providing information on possible risk factors for LTBI including demographic and socioeconomic details, presence of BCG scars, and degree of occupational exposure (year of training and active clinical/nursing practice in the TB or primary care service, job category, and nonmedical tuberculosis contact). Everyone identified as LTBI positive within the medical service was offered information on the published sensitivity and specificity of the tests, the potential value of these tests, and counselling on the risks and benefits of chemoprophylaxis. Within Samara, and Russia as a whole, chemoprophylaxis of household contacts is uniformly given. However, at present these IFN-γ tests are not licensed in the Russian Federation, and there are no guidelines on the use of these tests in the Russian Federation.

As the goal of the study was to recruit all clinical TB health care workers (HCWs) in Samara, no formal sample size determination was needed. TB staff were recruited from all TB clinics in the city; primary HCWs were recruited from a large polyclinic in Samara (Polyclinic 15). Polyclinics are the key primary care service providers and are organised on a per capita basis so that the polyclinic sampled would be representative of polyclinics across the city, particularly as we enrolled as close to all of the clinical staff as possible.

Blood samples (*n* = 630) from all participants were screened using a novel ex vivo IFN-γ release assay by drawing blood directly into QuantiFERON–TB Gold In-Tubes (Cellestis) containing ESAT-6 and CFP-10 antigens absent from BCG. Tubes were incubated at 37 °C overnight before centrifugation, and IFN-γ release was measured by ELISA as described by the manufacturer at the Samara Regional Tuberculosis Laboratory. All the assays performed met the manufacturer's quality control standards. Thirty plasma samples (4.8% of the total of 630 tested) were retested at the HPA Mycobacterium Reference Unit, London, and the concordance was 100%.

Statistical analysis was performed using the SPSS version 12 package (SPSS, http://www.spss.com). The difference of means was detected using Student's t-test. The difference between positivity rates among different groups was assessed using Pearson's Chi^2^ test (the groups were regarded as ordinal with increasing level of exposure from students to primary health care workers and TB workers groups). Risk factors for test positivity were defined using an odds ratio (OR) and relative risk (RR). Possible confounding effects between some of the variables, i.e., age and years of medical practice, were examined and accounted for. Only doctors and nurses were included in the risk factor analysis as “support staff,” and “others” (e.g., ward receptionists) were not recruited according to the original study protocol; they requested inclusion after the study had commenced as they felt they were also a heavily exposed group. To adjust for multiple covariates, we used an unconditional binary logistic regression model with IFN-γ test results as the outcome and covariates, such as markers of tuberculosis exposure. All variables included in the multivariate analysis were determined a priori based on estimation of their significance as epidemiological factors during the preliminary crude analysis (significant at *p* ≤ 0.05 and Pearson's correlation coefficient < 0.30) and biological plausibility. Model fit was evaluated using the Hosmer-Lemeshow test.

## Results

We recruited 630 individuals in total (598 directly recruited and 32 support workers who themselves requested testing but were not included in the final analysis) into the study; within the TB service 114/114 (100%) of all doctors and nurses; and 116/120 (96.7%) of those working in the polyclinic (Table 1). The prevalence of LTBI was determined in a low exposure group (*n* = 368) comprised of medical students in their final two years (245 students were invited to take part and 238 [97.1%] did so) and nonmedical students in their final year (130/130 [100%] participated) to establish the baseline prevalence, and whether the prevalence was broadly similar in both student groups. None of the participants had active TB, confirmed by absence of symptoms and recent chest radiography (all students and primary HCW within the previous 12 months). All TB staff underwent fluorographic and/or chest radiological screening biannually. None of the students or staff participants had experience working at TB facilities beyond short rotations (two three-week rotations in the preceding two years) in the TB service. Significantly, more medical students were born outside Samara Oblast (RR 2.1; 95% confidence interval [CI] 1.5–3.1) compared to nonmedical students, but no differences were detected between primary care and TB staff in this respect.

**Table 1:**
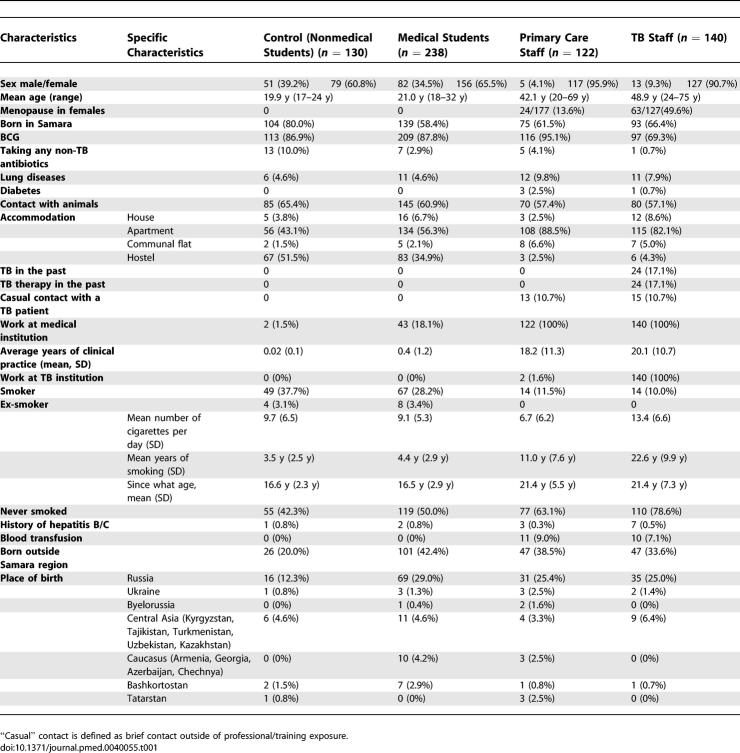
Baseline Characteristics of Student and Health-Care Groups

Baseline characteristics and absolute numbers of staff are given in Tables 1 and 2. Among health professionals the median number of years of clinical work was 18.2 and 20.1 y for primary care and TB staff, respectively.

**Table 2:**
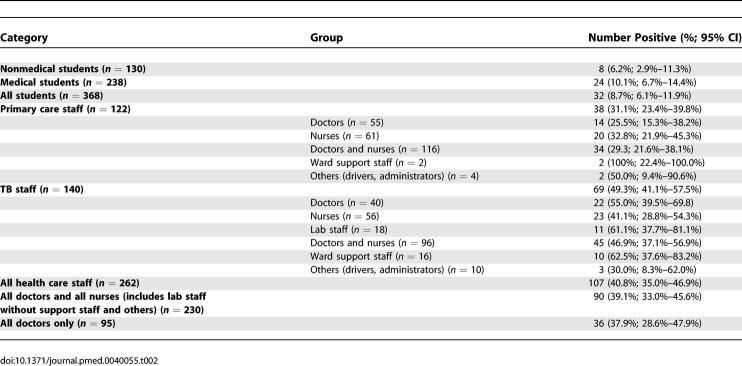
Number and Percentage Positive LTBI (IFN-γ Assay) by Exposure Group, Samara Oblast

The proportion of doctors and nurses among primary care workers and TB workers was similar; nonclinical staff were included in the descriptive analysis at this stage but as their recruitment was outside the planned strategy (they self-referred) they were not included in the univariate and multivariate analyses.

Although the LTBI rate in medical students (10.1%) appeared higher than in nonmedical students (6.2%) (presumably reflecting some exposure during their studies), this was not statistically significant, and none reported specific TB contact in their questionnaire. The majority of the participants had had BCG vaccination. Nevertheless, levels of positivity were relatively high (6.2% and 10.1%, respectively) for young people with apparently little contact with TB cases and not working in the area of TB control; it likely reflects general exposure in a high TB burden country. None of the students reported having an immunosuppressive condition or active TB, or were taking immunosuppressive drugs.

Across students, primary care workers, and TB staff, a trend for increased positivity was observed (χ^2^ for trend was 104.4, *p* < 0.001). The proportion of LTBI reflected by positive IFN-γ test was significantly higher in health care workers compared to the control group (all students), with 39.1% of all doctors and nurses being positive (versus 8.7% in students, RR 4.5 [95% CI 3.1–6.5]). Table 2 demonstrates the rates of IFN-γ positivity in different groups, and Table 3 compares the differences in proportions of IFN-γ-positive individuals between exposure groups.

**Table 3:**
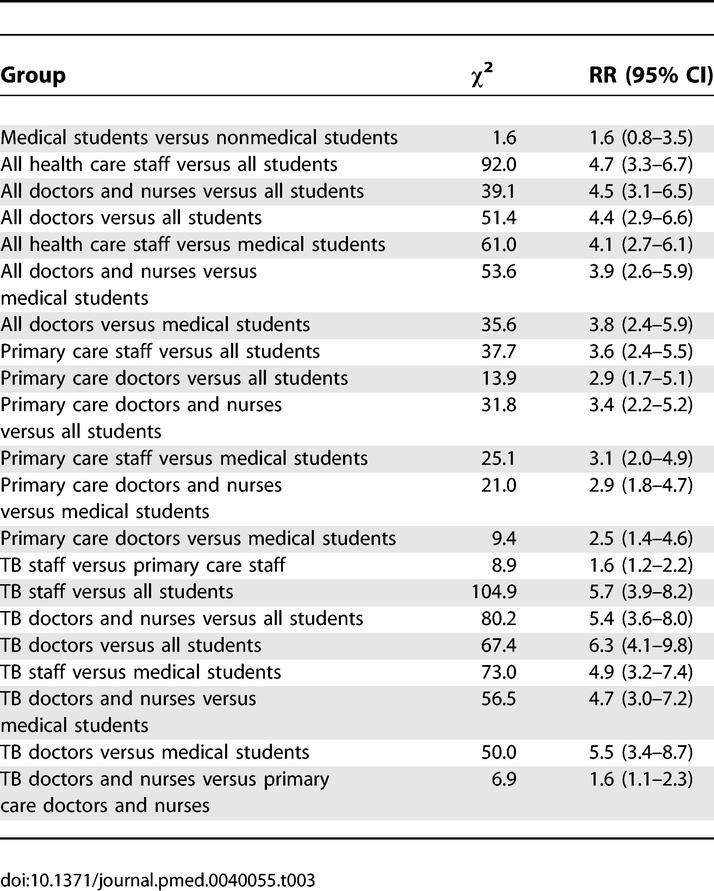
Relative Risk (%) and 95% Confidence Intervals of LTBI Positivity (IFN-γ) by Exposure Group, Samara Oblast

The mean IFN-γ concentration (standard deviation [SD]) in control students, medical students, primary care doctors and nurses, and TB doctors and nurses was 1.8 (1.9) IU/ml, 4.2 (5.0) IU/ml, 7.8 (7.3) IU/ml, and 3.0 (3.6) IU/ml, respectively. There was no correlation between the level of IFN-γ and age, nor level of IFN-γ and number of years of medical practice overall.

The proportion of people diagnosed with LTBI was significantly higher among primary health care doctors and nurses compared to the low-exposure group of medical students (29.3% versus 10.1%, RR 2.9 [95% CI 1.8–4.7]) and in TB doctors and nurses compared to primary health care workers or medical students (46.9% versus 29.3%, RR 1.6 [95% CI 1.1–2.3] and versus 10.1%, RR 4.7 [95% CI 3.0–7.2]).

Statistically significant differences in the proportions of individuals with LTBI were found between medical students, primary doctors, and TB doctors specifically as well as between all students, all primary care staff, and all TB clinical staff, reflecting the gradient of increased TB exposure (Table 3).

We also identified risk factors for LTBI among students, doctors, and nurses. The univariate analysis demonstrated that among all students there were no significant predictors of positivity. Among doctors and/or nurses (working in general care and the TB service), age over 35 years, TB in the past, work in a TB clinic, and more than five years in medical practice were associated with having LTBI on univariate analysis (OR 3.1, 95% CI 1.5–6.4; OR 2.8, 95% CI 1.1–7.0; OR 2.6, 95% CI 1.5–4.4; and OR 2.5, 95% CI 1.4–5.0 and correlation coefficients of 0.136, *p* = 0.002; 0.148, *p* = 0.025; 0.225, *p* = 0.001; and 0.114, *p* = 0.003, respectively).

Gender was not associated with a positive test result. Multivariate analysis demonstrated that work in a TB clinic was the only significant risk factor for LTBI (OR 1.9, 95% CI 1.1–3.5, *p* = 0.031).

## Discussion

This is the first study, to our knowledge, to directly establish rates of TB infection in HCWs in any former Soviet Union country using novel direct in-tube in vitro IFN-γ assays. QuantiFERON–TB Gold In-Tube assays are as sensitive and more specific than traditionally used PPD tests, varying between 59% and 89%, and 53% and 100%, respectively [10–15]. Nearly everyone who was invited to participate in the study did so, reflecting an interest in and a level of awareness of the problem of TB.

In vitro IFN-γ assays are likely to be of particular value in determining LTBI in former Soviet Union countries because of the practice of giving multiple BCG vaccinations, previous repetitive TSTs in childhood, and an existing annual screening policy for TB in health care workers [16].

The study supports our hypothesis that there was a high prevalence of LTBI in staff within the TB service (i.e., a high-exposure group), including those working in the laboratory compared to students (low-exposure group), and clinical staff (doctors and nurses) working in primary practice (intermediate exposure). A high proportion of self-referred support staff were also LTBI positive. An increasing proportion of infected individuals were seen with increasing occupational health exposure to TB.

The multivariate analysis demonstrated that work in a TB service was the only important risk factor for LTBI, reflecting extensive exposure to M. tuberculosis. Our study supports previous evidence that results of ESAT-6 and CFP-10 based IFN-γ tests results are not affected by BCG vaccination [11], particularly relevant in a country where repetitive BCG vaccination is national policy.

Our results are consistent with recently published results of two studies in HCWs from a high- (India) and a low- (Japan) incidence country [14,17,18], but these were in single institutions. Similar risk factors for infection were demonstrated at a hospital in India using these assays [17] and in previous skin test surveys of HCWs in India using PPD, which were likely confounded by BCG vaccination.

The study emphasises the importance of studying LTBI in all HCW, as infection rates were high in an intermediate-risk group (primary care) as well as in a high-risk group (TB health workers); as TB patients are treated in a distinct vertical TB service, we were able to clearly define HCW groups with different TB exposure and contrast these with students. Although not strictly a sample-based strategy, the present group of students, doctors, nurses, and laboratory staff, in particular those working in the TB service, are at increased risk of infection, suggesting that appropriate preventive strategies and/or staff health monitoring should be undertaken. HCWs in this region of Russia are likely to be representative of Russia as a whole, as the organization of the health system in Samara is almost identical to that of Russia and is similar to all countries of the former Soviet Union. Similarly, the TB incidence and prevalence is close to the national average for Russia [4].

The IFN-γ-based assays appear to offer rapid in vitro testing requiring a single screening visit, which is less subjective than skin testing and can be repeated as an annual occupational screening. Improved institutional cross-infection strategies and subsequent preventive therapy for those infected will be of value to health professionals and their patients. However, the reagent cost of the test is high, requiring an appropriate laboratory infrastructure, and the samples need to be processed within a short period of time.

The findings in this study should also help to inform occupational health policy in countries such as Russia and the United States, where there is an annual screening of health care staff for TB using chest X-ray (Russia), and Mantoux testing (United States), or no clear annual policy (such as the United Kingdom). IFN-γ assays demonstrate good performance in serial testing [19] and therefore could have a useful role in regular screening of HCWs and students with a high risk of LTBI, particularly where recent infection is demonstrated. Nevertheless, larger cohort studies are needed to assess the value of these tests results, risks of active TB development in positive individuals, and effectiveness of preventive therapy based on IFN-γ test results.

The key question globally will be how these results are used. In the Russian context the results will be of greatest value in modifying institutional infection control and occupational health policies, which have traditionally been weak. Negative-pressure facilities are not usually available in Russian hospitals, including those centres dealing exclusively with TB, unlike the United States or most parts of Western Europe; the main feature of TB control is the use of ultraviolet germicidal radiation and daily surface disinfection. A similar practice operates in the laboratories. Except in winter, windows are opened to provide ventilation.

Individually, preventive therapy given to those with a positive tuberculin test substantially decreases the risk of development of active TB [20]. However, there has been no trial to demonstrate the efficacy and cost-effectiveness of administration of preventive therapy based on IFN-γ test results, although it would be reasonable to suppose that there would be some benefit. For example, chemoprophylaxis following positive TSTs outside Russia is limited largely to younger age groups. In Russia it is widely practised for children and for close contacts of patients with active TB (without conducting a TST, because of the high probability of false-positive results in adults). All HCW staff are radiologically screened for TB annually with referral to the TB service for chemoprophylaxis or full treatment as appropriate. Within the TB service, isoniazid prophylaxis is not widely practised, as there is an argument that it would be of limited value due to the continued exposure to new cases.

The value of chemoprophylaxis in Samara will be reduced by the high prevalence of isoniazid and multidrug-resistant tuberculosis reported there (as these tests cannot distinguish between infection with drug sensitive and resistant organisms) [3,4] and elsewhere in Russia [21]; nevertheless, the majority of individuals will still have been infected with susceptible organisms, and it is not known how extensive drug resistance is across the Russian population, as well-designed drug resistance surveys have only been reported for a few regions of the Russian Federation. Indeed a study in Orel, Russia demonstrated a relatively low incidence of drug resistance [22]. Furthermore, chemoprophylaxis may be more effective in Samara and comparable regions for addressing latent infection than active disease due to the small bacterial organism load where the relevant population of drug resistant organisms are a small proportion of a low number of bacteria; these organisms may be destroyed or controlled by the individual's cell immune system. There is some anecdotal evidence for this in that there have been very few active cases of TB reported in TB contacts in Samara, given empirical chemoprophylaxis.

In contrast, in the United States, guidelines from the Centers for Disease Control (CDC) [23] suggest that the Food and Drug Administration (FDA) approved QuantiFERON–TB Gold assay can be used wherever Mantoux testing would be employed, with the assumption that chemoprophylaxis will be applied in the same way and with similar efficacy. This study, using the QuantiFERON–TB Gold In-Tube method (which is awaiting FDA approval) will help determine policy in those countries where BCG vaccination is used (the majority). Although studies have demonstrated the benefits of using IFN-γ tests in low-incidence countries where a new positive result appears to demonstrate recent infection [24], in middle- to high-incidence countries the high number of positive individuals may make wide scale chemoprophylaxis unworkable. Ultimately, the ability to deliver an effective and appropriate chemoprophylaxis strategy is essential if these tests are to be of real clinical and public health benefit.

Further economic studies are also needed to evaluate the cost-effectiveness of using these tests in middle-income high-incidence countries as well as low-incidence countries such as the United Kingdom and the United States.

## Abbreviations

BCG: bacille Calmette-Guérin
CI: confidence interval
HCW: health care workers
IFN-γ: gamma-interferon
LTBI: latent tuberculosis infection
OR: odds ratio
PPD: tuberculin
RR: relative risk
TB: tuberculosis
TST: tuberculin skin test

## References

[pmed-0040055-b001] Tufariello JM, Chan J, Flynn JL (2003). Latent tuberculosis: Mechanisms of host and bacillus that contribute to persistent infection. Lancet Infect Dis.

[pmed-0040055-b002] Spradling P, Nemtsova E, Aptekar T, Shulgina M, Rybka L (2002). Anti-tuberculosis drug resistance in community and prison patients, Orel Oblast, Russian Federation. Int J Tuberc Lung Dis.

[pmed-0040055-b003] Ruddy M, Balabanova Y, Graham C, Fedorin I, Malomanova N (2005). Rates of drug resistance and risk factor analysis in civilian and prison patients with tuberculosis in Samara Region, Russia. Thorax.

[pmed-0040055-b004] Drobniewski F, Balabanova Y, Nikolayevskyy V, Ruddy M, Kuznetzov S (2005). Drug resistant tuberculosis, clinical virulence and the dominance of the Beijing strain family in Russia. JAMA.

[pmed-0040055-b005] Ministry of Health, Russian Federation (2003). On improvement of TB control activities in the Russian Federation. Prikaz number 109.

[pmed-0040055-b006] Dimitrova B, Hutchings A, Atun R, Drobniewski F, Marchenko G (2005). Increased risk of tuberculosis among health care workers in Samara Oblast, Russia: Analysis of notification data. Int J Tuberc Lung Dis.

[pmed-0040055-b007] Andersen P, Munk ME, Pollock JM, Doherty TM (2000). Specific immune-based diagnosis of tuberculosis. Lancet.

[pmed-0040055-b008] Pai M, Kalantri S, Dheda K (2006). New tools and emerging technologies for the diagnosis of tuberculosis: Part II. Active tuberculosis and drug resistance. Expert Rev Mol Diagn.

[pmed-0040055-b009] Lalvani A (2004). Counting antigen-specific T cells: A new approach for monitoring response to tuberculosis treatment?. Clin Infect Dis.

[pmed-0040055-b010] Dheda K, Chang JS, Kim LU, Huggett JF, Johnson MA (2005). Interferon gamma assays for tuberculosis. Lancet Infect Dis.

[pmed-0040055-b011] Lalvani A, Pathan AA, Durkan H, Wilkinson KA, Whelan A (2001). Enhanced contact tracing and spatial tracking of Mycobacterium tuberculosis infection by enumeration of antigen-specific T cells. Lancet.

[pmed-0040055-b012] Lalvani A, Richeldi L, Kunst H (2005). Interferon gamma assays for tuberculosis. Lancet Infect Dis.

[pmed-0040055-b013] Mazurek GH, LoBue PA, Daley CL, Bernardo J, Lardizabal AA (2001). Comparison of a whole-blood interferon gamma assay with tuberculin skin testing for detecting latent Mycobacterium tuberculosis infection. JAMA.

[pmed-0040055-b014] Mori T, Sakatani M, Yamagishi F, Takashima T, Kawabe Y (2004). Specific detection of tuberculosis infection: An interferon-gamma-based assay using new antigens. Am J Respir Crit Care Med.

[pmed-0040055-b015] Whalen CC (2005). Diagnosis of latent tuberculosis infection: Measure for measure. JAMA.

[pmed-0040055-b016] Drobniewski FA, Tayler E, Ignatenko N, Paul J, Connolly M (1996). TB in Siberia 2- diagnosis, chemoprophylaxis and treatment. Tuber Lung Disease.

[pmed-0040055-b017] Pai M, Gokhale K, Joshi R, Dogra S, Kalantri S (2005). Mycobacterium tuberculosis infection in health care workers in rural India: Comparison of a whole-blood interferon gamma assay with tuberculin skin testing. JAMA.

[pmed-0040055-b018] Pai S, Esen N, Pan X, Musser JM (1997). Routine rapid *Mycobacterium* species assignment based on species-specific allelic variation in the 65-kilodalton heat shock protein gene (hsp65). Arch Pathol Lab Med.

[pmed-0040055-b019] Pai M, Joshi R, Dogra S, Mendiratta DK, Narang P (2006). Serial testing of health care workers for tuberculosis using interferon-γ assay. Am J Respir Crit Care Med.

[pmed-0040055-b020] American Thoracic Society and the Centers for Disease Control and Prevention (2000). Targeted tuberculin testing and treatment of latent tuberculosis infection. Am J Respir Crit Care Med.

[pmed-0040055-b021] World Health Organization/ International Union Against Tuberculosis and Lung Disease (WHO/IUALTD) Global Project on Anti-Tuberculosis Drug Surveillance (2004). Anti-tuberculosis drug resistance in the world: Third global report/the WHO/IUALTD Global Project on Anti-Tuberculosis Drug Surveillance, 1999–2002.

[pmed-0040055-b022] Dewan PK, Arguin PM, Kiryanova H, Kondroshova NV, Khorosheva TM (2004). Risk factors for death during tuberculosis treatment in Orel, Russia. Int J Tuberc Lung Dis.

[pmed-0040055-b023] Mazurek GH, Jereb J, Lobue P, Iademarco MF, Metchock B (2005). Guidelines for using the QuantiFERON-TB Gold test for detecting Mycobacterium tuberculosis infection, United States. MMWR Recomm Rep.

[pmed-0040055-b024] Doherty TM, Demissie A, Olobo J, Wolday D, Britton S (2002). Immune responses to the Mycobacterium tuberculosis-specific antigen ESAT-6 signal subclinical infection among contacts of tuberculosis patients. J Clin Microbiol.

